# Rescue of the Friedreich Ataxia Knockout Mutation in Transgenic Mice Containing an *FXN-EGFP* Genomic Reporter

**DOI:** 10.1371/journal.pone.0093307

**Published:** 2014-03-25

**Authors:** Joseph P. Sarsero, Timothy P. Holloway, Lingli Li, David I. Finkelstein, Panos A. Ioannou

**Affiliations:** 1 Cell and Gene Therapy, Murdoch Childrens Research Institute, Royal Children’s Hospital, Parkville, Victoria, Australia; 2 Department of Paediatrics, The University of Melbourne, Royal Children’s Hospital, Parkville, Victoria, Australia; 3 Florey Institute of Neuroscience and Mental Health, The University of Melbourne, Victoria, Australia; University of South Florida, United States of America

## Abstract

Friedreich ataxia (FRDA) is an autosomal recessive disorder characterized by neurodegeneration and cardiomyopathy. The presence of a GAA trinucleotide repeat expansion in the first intron of the *FXN* gene results in the inhibition of gene expression and an insufficiency of the mitochondrial protein frataxin. We previously generated BAC-based transgenic mice containing an *FXN-EGFP* genomic reporter construct in which the EGFP gene is fused in-frame immediately following the final codon of exon 5a of the human *FXN* gene. These transgenic mice were mated with mice heterozygous for a knockout mutation of the murine *Fxn* gene, to generate mice homozygous for the *Fxn* knockout mutation and hemizygous or homozygous for the human transgene. Rescue of the embryonic lethality that is associated with homozygosity for the *Fxn* knockout mutation was observed. Rescue mice displayed normal behavioral and histological parameters with normal viability, fertility and life span and without any signs of aberrant phenotype. Immunoblotting demonstrated the production of full-length frataxin-EGFP fusion protein that appears to act as a bifunctional hybrid protein. This study shows frataxin replacement may be a viable therapeutic option. Further, these mice should provide a useful resource for the study of human *FXN* gene expression, frataxin function, the evaluation of pharmacologic inducers of *FXN* expression in a whole-animal model and provide a useful source of cells for stem cell transplantation studies.

## Introduction

Friedreich ataxia (FRDA; OMIM 229300) is an autosomal recessive disorder characterized by neurodegeneration and cardiomyopathy. It is the most common form of hereditary ataxia with an incidence of approximately 1 in 50,000 in Caucasian populations [1]. Approximately 98% of individuals with FRDA are homozygous for an expansion of a GAA trinucleotide repeat sequence within the first intron of the *FXN* gene. The remaining individuals are compound heterozygotes for a GAA expansion and a point mutation, deletion and/or insertion. Pathogenic GAA expansion alleles are in the size range of 60 to more than 1300 repeats. The presence of a GAA repeat expansion results in the inhibition of *FXN* gene expression, reduced levels of full length *FXN* transcript and an insufficiency of the mitochondrial protein frataxin [2]–[5].

Frataxin deficiency results in mitochondrial dysfunction including the loss of iron-sulfur cluster (ISC)-containing enzymes, increased oxidative damage and mitochondrial iron accumulation [6]–[8]. Frataxin has been implicated to play an important role in the early stages of iron-sulfur cluster biogenesis by direct interaction with ISU-type proteins [9]–[12]. The main sites of pathology include the large sensory neurons of the dorsal root ganglia and the neurons of the dentate nucleus of the cerebellum [13], [14].

The mechanism by which the GAA expansion results in reduced *FXN* gene expression is not clear. It was initially suggested that the GAA repeat expansion may form an unusual and stable triple helical non-B DNA structure or DNA/RNA hybrid that impedes transcription elongation [4], [5], [15]. It is now evident that the GAA repeat expansion generates a heterochromatin-mediated gene silencing effect [16], [17]. Repressive changes in both DNA methylation and histone modification have been described [17]–[24].

We previously reported the identification of a 188 kb Bacterial Artificial Chromosome (BAC) clone (RP11-265B8) containing exons 1–5 b of the human *FXN* locus and extensive flanking regions both upstream and downstream of the gene [25]. The clone contains a (GAA)_6_ sequence in the region that undergoes expansion within the first intron of the *FXN* gene. We demonstrated that the genomic insert is able to successfully complement the embryonic lethal phenotype of homozygous *Fxn* knockout mice, indicating that key regulatory elements required for normal expression of the *FXN* gene are present within this clone [26].

Key control regions can be located large distances from the genes that they regulate and the normal location and spacing of many regulatory elements is crucial to facilitate physiological gene expression patterns. Genomic reporters, in which a reporter fusion is made to a gene in the context of its entire genomic locus on a BAC clone, preserve the positional relationships of regulatory elements in the surrounding chromosomal region, and facilitate the recapitulation of normal gene expression patterns. The RP11-265B8 BAC clone was used for the generation of an *FXN-EGFP* genomic reporter construct in which the EGFP gene was fused in-frame immediately following the final codon of exon 5a of the human *FXN* gene [25]. The construct was shown to drive the expression of EGFP from the regulatory elements of the *FXN* locus [27] with the frataxin-EGFP fusion protein targeted to the mitochondria [25], [28].

We previously described the utilization of the *FXN-EGFP* genomic reporter construct for the generation of transgenic mice lines that provided a sensitive and convenient *in vivo* assay for human *FXN* gene expression [29]. Production of full-length frataxin-EGFP fusion protein was evident in the transgenic mice. EGFP expression was observed as early as day E3.5 of development and most tissues of adult transgenic mice were fluorescent. Analysis by flow cytometry of various cell types after enzymatic tissue dissociation allowed a quantitative assessment of *FXN* gene expression. There was a direct correlation between gene dosage and EGFP expression levels [29]. These transgenic mice have served as a valuable tool for the examination of spatial and temporal aspects of *FXN* gene expression, and tissues from these mice have been used for stem cell transplantation studies [30].

In this study we utilized the previously developed tools to generate a new transgenic mouse line that has the *FXN-EGFP* genomic reporter in a homozygous *Fxn* knockout background. This study demonstrates that the *FXN-EGFP* genomic reporter is able to rescue the embryonic lethal phenotype and compensate for the loss of endogenous murine frataxin.

## Materials and Methods

### Ethics Statement

All animal procedures were conducted under the approval of the Murdoch Childrens Research Institute Animal Experimentation Ethics Committee and the Florey Institute of Neuroscience and Mental Health Animal Ethics Committee. All efforts were made to minimize suffering.

### Mouse Maintenance

Mice were housed in Tecniplast IVC cages with irradiated Fibrecycle bedding, with seeds and cardboard homes as environmental enrichment, with 14 hours light, 10 hours dark, at 23°C and 44–65% humidity. The mice were given a diet of irradiated Ridley Agri Mouse breeder cubes and standard autoclaved drinking water. Mouse strains have a congenic C57BL/6 background.

Mice were checked immediately after birth for any gross abnormalities and then daily for growth activity and general behavior. Body condition, breathing, mobility, movement and gait were observed daily. Mice were monitored for signs of distress including severe weight loss, soft feces, ruffled fur, hunched posture, lethargy, vocalization, convulsions, inactivity, and paralysis. Mice showing any signs of distress were humanely euthanized using isoflurane (Provet, Heatherton, Victoria, Australia) and cervical dislocation. Mice were maintained up to a maximum age of 18 months and then humanely euthanized. No mice were permitted to expire without intervention.

### Genotype Analysis

Genomic DNA was extracted from mouse tail biopsies as previously described [31]. Mice were screened with primers EGFP-probe-F (5′-ATGGTGAGCAAGGGCGAGGAGCTGTT-3′) and EGFP-probe-R (5′-CTGGGTGCTCAGGTAGTGGTTGTC-3′), that amplify a 615 bp region of the EGFP gene in the *FXN-EGFP* genomic reporter construct [29]. The thermal profile was 94°C for 10 minutes, 30 cycles of 94°C for 30 seconds, 60°C for 30 seconds, and 72°C for 1 minute, and a final cycle at 72°C for 10 minutes. The identification of wild type or knockout exon 4 of the murine *Fxn* gene was performed with multiplex PCR as previously described [32].

### Western Blot Analysis

Lysates were prepared from tissues by homogenization in three volumes of lysis buffer [100 mM Tris (pH 9.0), 2% SDS, 15% glycerol, and protease inhibitor cocktail (Sigma-Aldrich, St. Louis, MO, USA)] using a Polytron homogenizer (Kinemetica, Switzerland), followed by sonication for 15 seconds on ice and centrifugation (10,000 g for 15 minutes at 4°C). Protein concentration was determined with a DC Protein Assay kit (Bio-Rad, Hercules, CA, USA). 200 μg of protein was electrophoresed on 12% SDS-polyacrylamide gels followed by electrophoretic transfer onto a nitrocellulose membrane (Hybond-C, Amersham, Little Chalfont, UK). The position of protein bands was estimated using pre-stained molecular weight markers (Bio-Rad) and the efficiency of transfer was revealed by staining the membrane with Ponceau S. Immunoblotting and visualization were performed using the Lumi-Light^PLUS^ Western Blotting kit (Roche). Human frataxin was detected with the anti-frataxin monoclonal antibody 2FRA-1G2 (Chemicon, Temecula, CA, USA) at 1∶5,000 dilution for 24 hours at 4°C. EGFP was detected with rabbit anti-GFP polyclonal antibody (BD Living Colors A.v. Peptide Antibody, BD Biosciences Clontech, Palo Alto, CA, USA) at 1∶10,000 dilution for 1 hour at 4°C. Horseradish peroxidase-conjugated rabbit anti-mouse immunoglobulin and mouse anti-rabbit immunoglobulin (Roche) were used as secondary antibodies for 30 minutes at room temperature. Recombinant GFP protein was used as a positive control (BD Biosciences Clontech).

### Locomotor Function

Locomotor function was assessed on four male and four female mice of each specified genotype (total of 16 mice) at 12 months of age with a Rotarod device (3.6 cm rod diameter, Mouse-Rota MK-2; Monash University, Australia). All mice were initially trained in two ramping sessions of 3–30 rpm over a five minute time period and a single session of constant 16 rpm for five minutes. The test procedure was performed three times at a constant speed of 16 rpm for a maximum of three minutes. A rest period of at least 20 minutes was allowed between each session [33]. Statistical analyses were performed by using ANOVA with replication.

### Histology

Histopathology was performed on the same cohort of mice used for rotarod analysis at 12 months of age. Mice were anesthetized with sodium pentobarbitone (100 mg/kg) via intraperitoneal injection. Following anesthesia mice were transcardially perfused with phosphate buffered saline followed by Bouin’s fixative (4% paraformaldehyde and 0.2% picric acid; 40 ml/animal). Brains, spinal cords and dorsal root ganglia (DRG) were dissected out of each animal and placed in Bouin’s fixative. All tissue samples were postfixed for 12 hours in Bouin’s fixative at 4°C. The tissue samples were processed and embedded in Paraplast Plus wax tissue embedding medium (Sigma-Aldrich). Cross sections (5 μm) of each tissue were prepared and representative sections stained with haematoxylin and eosin.

## Results and Discussion

### Complementation of *Fxn* Knockout

We have previously demonstrated that the 188 kb human genomic DNA fragment in BAC RP11-265B8 is able to rescue the embryonic lethality of the homozygous murine *Fxn* gene knockout when the transgene was in hemizygous or homozygous form. The rescue mice displayed normal behavioral and biochemical parameters indistinguishable from normal mice [26]. The DNA fragment used in these studies contains all sequences necessary for the correct expression of the human *FXN* gene. This is the smallest human genomic fragment shown to complement the *Fxn* knockout mutation and complement for the loss of endogenous murine frataxin.

**Figure 1:**
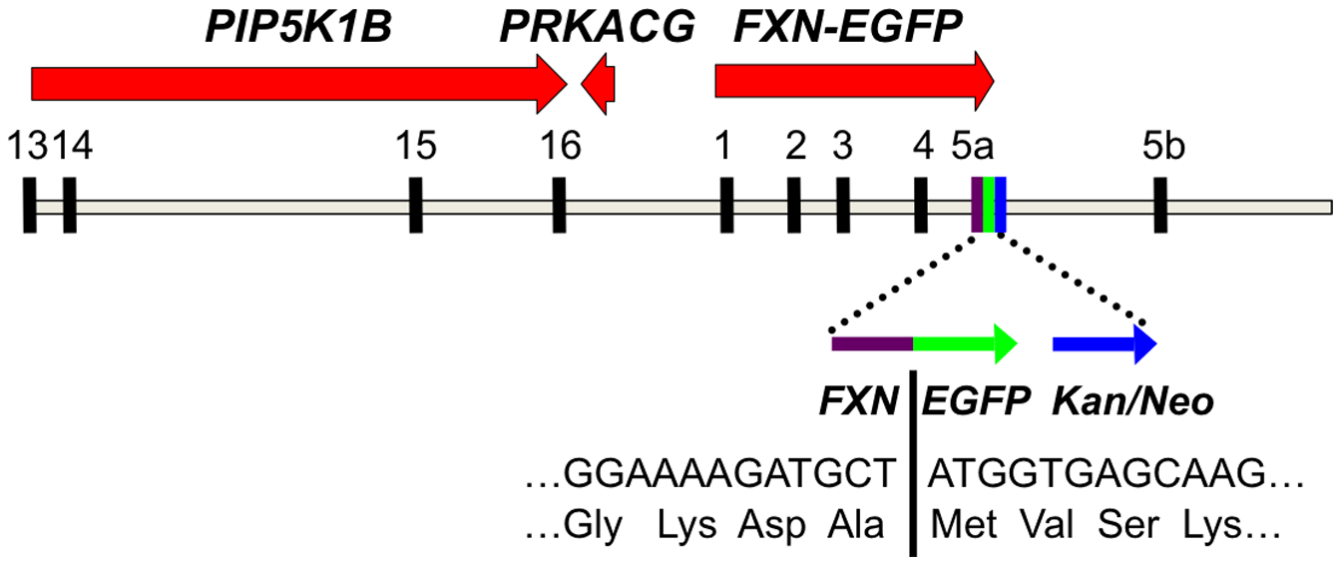
*FXN-EGFP* genomic reporter construct. Diagrammatic representation of the BAC genomic DNA fragment containing the *FXN-EGFP* genomic reporter construct. The sequence includes exons 13–16 of the *PIP5K1B* gene and the *PRKACG* gene upstream of the *FXN* locus, and about 23 kb of additional sequence downstream of exon 5b. The exon 5a–EGFP–Kan/Neo region is shown in greater detail.

We previously generated an *FXN-EGFP* genomic reporter fusion by using homologous recombination [34] to insert an EGFP-Kan/Neo cassette in-frame immediately following the final codon of exon 5a of the normal human *FXN* gene present on the RP11-265B8 BAC clone [25]. This construct encodes a frataxin-EGFP fusion protein that is expressed via the endogenous *FXN* promoter and under the control of regulatory elements surrounding the intact *FXN* locus [27]. The modified genomic insert from the BAC clone (**Figure 1**) was isolated from most of the vector sequence by digestion at unique sites with *Asc*I and *Bsi*WI and purified linear DNA was used to generate transgenic mice by pronuclear microinjection of C57BL/6 fertilized oocytes [29].

**Figure 2:**
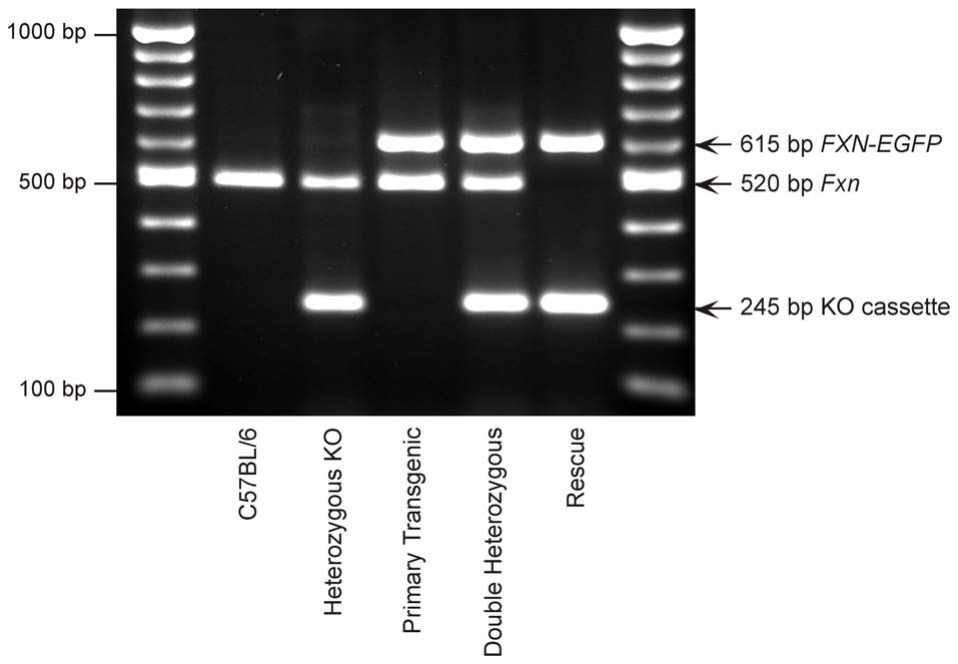
PCR-based genotype analysis. The generation of transgenic mice containing the *FXN-EGFP* genomic reporter construct in a murine *Fxn* knockout background was analyzed by PCR. The 615 bp band corresponds to the EGFP sequence in the *FXN-EGFP* transgene; the 520 bp band corresponds to the endogenous wild type murine *Fxn* gene; the 245 bp band corresponds to the *Fxn* knockout allele. C57BL/6, wild-type mouse (*Fxn* ^+/+^); Heterozygous KO, (*Fxn* ^+/−^); Primary transgenic, (*FXN-EGFP* ^+^, *Fxn* ^+/+^); Double heterozygous, (*FXN-EGFP* ^+^, *Fxn* ^+/−^); Rescue, (*FXN-EGFP* ^+^, *Fxn* ^−/−^).

In order to determine if the human *FXN-EGFP* genomic fragment was able to complement the endogenous murine *Fxn* gene, we introduced the transgene into a homozygous *Fxn* knockout background. One of our previously established transgenic mouse lines containing three copies of the *FXN-EGFP* genomic reporter in a single integration site [29] was first crossed with heterozygous *Fxn* knockout mice (*Fxn* ^+/−^) [32] on a C57BL/6 background. Multiplex PCR screening of offspring identified ‘double heterozygous’ mice (*FXN-EGFP* ^+^, *Fxn* ^+/−^), which were hemizygous for the *FXN-EGFP* transgene and heterozygous for the *Fxn* knockout (**Figure 2**). Twenty-two double heterozygous mice were identified from a total of 97 mice which agrees with the expected frequency of 1/4 for this genotype.

The double heterozygous mice were then inter-crossed with each other. No animals of the genotype *FXN-EGFP* ^−^, *Fxn* ^−/−^ were recovered, consistent with the embryonic lethal phenotype displayed by homozygous *Fxn* knockout mice. Sixteen mice from a total of 95 mice were identified with a complete absence of the endogenous *Fxn* gene and the presence of the human *FXN-EGFP* transgene (*FXN-EGFP* ^+^, *Fxn* ^−/−^ or *FXN-EGFP* ^+/+^, *Fxn* ^−/−^). This number approximates the expected frequency of rescue mice which is 3/15 live births. The presence of the BAC-derived genomic fragment containing the *FXN-EGFP* reporter construct was able to rescue the *Fxn* knockout mutation (**Figure 2**).

Competitive PCR [26], [29] was used to determine the copy number of the human transgene in the rescue animals. Mice with three copies of the transgene (hemizygous) and mice with six copies of the transgene (indicating homozygosity for the transgene insertion) have been identified (data not shown). The BAC-derived genomic *FXN* fragment containing the *FXN-EGFP* genomic reporter fusion is able to complement the *Fxn* knockout mutation in homozygous or hemizygous form, thus demonstrating the functional integrity of at least one copy of the human *FXN* locus in the transgenic mouse lines.

Rescue mice have been interbred to establish purely rescued colonies. All offspring are of the genotypes *FXN-EGFP* ^+^, *Fxn* ^−/−^ or *FXN-EGFP* ^+/+^, *Fxn* ^−/−^. In the first intercross of mice that were hemizygous for the *FXN-EGFP* transgene and heterozygous for the *Fxn* knockout (*FXN-EGFP* ^+^, *Fxn* ^−/−^) 18 of 61 mice were identified as homozygous for the *FXN-EGFP* transgene (*FXN-EGFP* ^+/+^, *Fxn* ^−/−^) which approximates the expected frequency of 1/3 live births.

### Analysis of Frataxin-EGFP Fusion Protein

**Figure 3:**
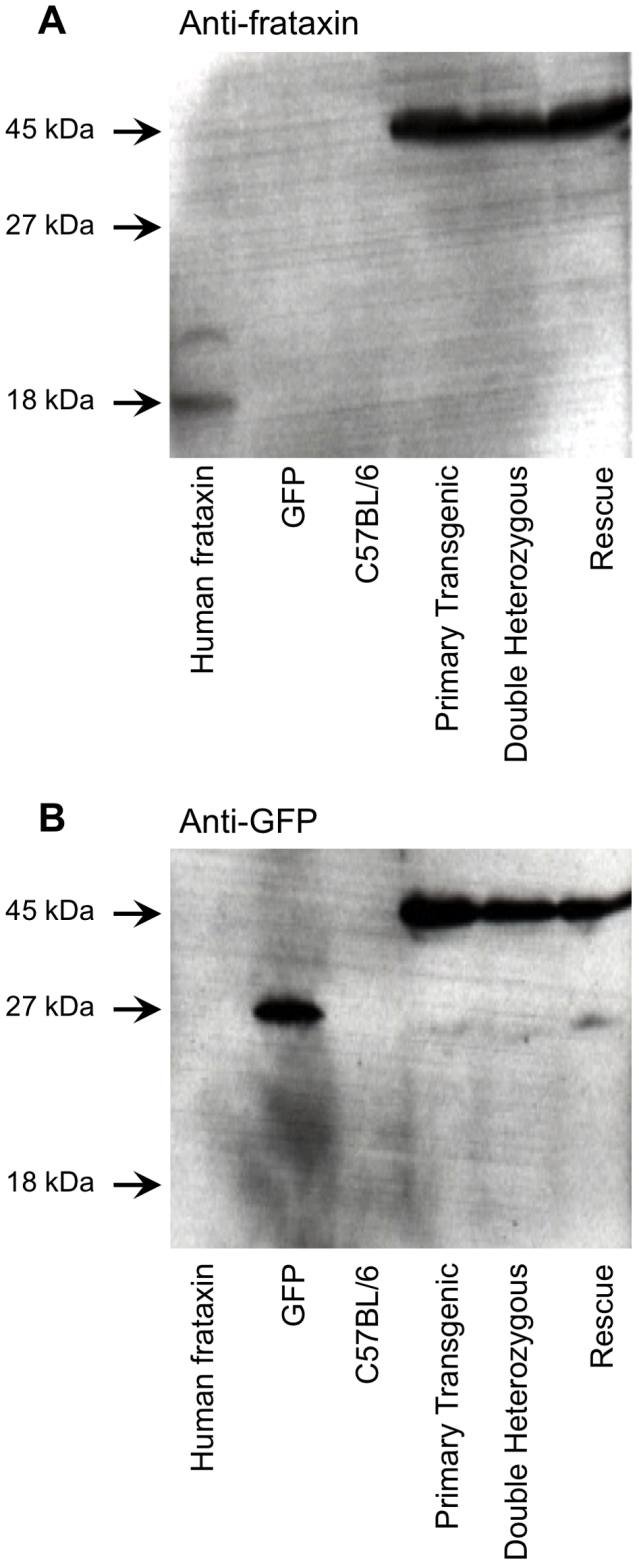
Detection of frataxin-EGFP fusion protein. Western blot analysis was carried out on protein extracts from liver tissue of *FXN-EGFP* transgenic mice and C57BL/6 control mice. Identical blots were probed with either anti-human frataxin monoclonal antibody (A) or with anti-GFP polyclonal antibody (B). A liver cell lysate from a transgenic mouse expressing human frataxin [26] was used as a positive control for the anti-frataxin antibody, and purified recombinant GFP was used as a positive control for the anti-GFP antibody. Both antibodies detected a protein of the expected size of the full-length frataxin-EGFP fusion (45 kDa) in transgenic mice. Cross-reaction of the anti-human frataxin antibody with murine frataxin was not evident under the experimental conditions used. C57BL/6, wild-type mouse; Primary transgenic, (*FXN-EGFP* ^+^, *Fxn* ^+/+^); Double heterozygous, (*FXN-EGFP* ^+^, *Fxn* ^+/−^); Rescue, (*FXN-EGFP* ^+^, *Fxn* ^−/−^).

We previously showed production of full-length frataxin-EGFP fusion protein in transgenic mice harboring the *FXN-EGFP* genomic reporter construct [29]. To analyze the expression of the *FRDA-EGFP* genomic reporter construct in the newly generated transgenic mouse lines total cellular protein was prepared from liver samples of the previously established *FXN-EGFP* primary transgenic mice (*FXN-EGFP* ^+^, *Fxn* ^+/+^) [29], and from double heterozygous mice (*FXN-EGFP* ^+^, *Fxn* ^+/−^) and rescue mice (*FXN-EGFP* ^+^, *Fxn* ^−/−^) and subjected to Western blot analysis using antibodies specific to human frataxin and to GFP. The processed mature mitochondrial form of human frataxin is 18 kDa in size and EGFP is 27 kDa. Both antibodies detected a band of 45 kDa in all transgenic mouse lines, which corresponds to the size expected of the full-length frataxin-EGFP fusion protein in which the 80 amino acid mitochondrial targeting sequence at the amino-terminus of frataxin has been cleaved (**Figure 3**). These results demonstrate the production of full-length frataxin-EGFP fusion protein under the control of the natural regulatory elements surrounding the human *FXN* gene present on the genomic DNA fragment.

### Phenotype Analyses

No gross phenotypic or behavioral differences were apparent between the *FRDA-EGFP* rescue mice and normal littermates. No discernible difference in life expectancy was evident. Mice were maintained up to a maximum age of 18 months. Body weights were similar to those of normal sex and age matched mice (data not shown).

**Figure 4:**
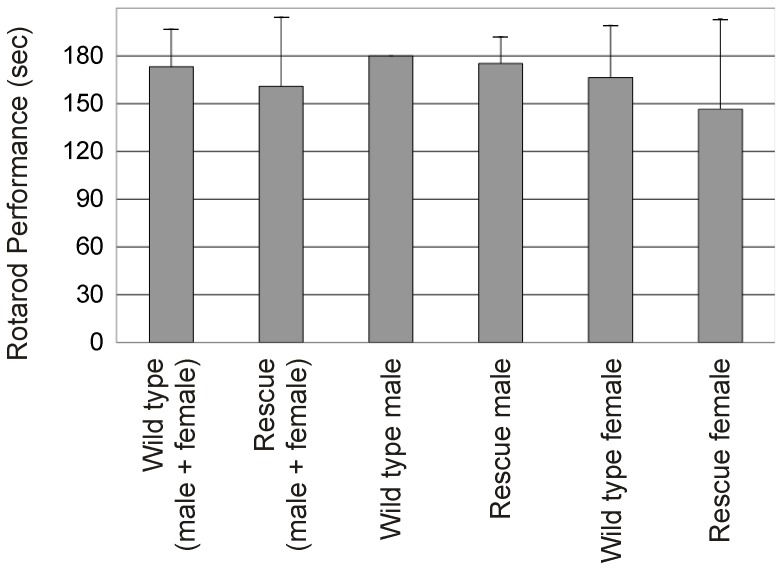
Rotarod analysis. Locomotor function was assessed by rotarod analysis. Performance is rated as time on rotarod with 180 seconds maximum. Four male and four female wild type and *FXN-EGFP* rescue mice (*FXN-EGFP* ^+^, *Fxn* ^−/−^) at 12 months of age were analyzed. Error bars represent standard error of the mean.

The main clinical features of FRDA include progressive limb and gait ataxia, features also displayed by conditional knockout and YAC-based GAA repeat expansion mouse models of FRDA [8], [35]. A cohort of wild type and *FXN-EGFP* rescue mice (*FXN-EGFP* ^+^, *Fxn* ^−/−^) were examined for locomotor function by performing rotarod tests at 12 months of age. No statistically significant difference was observed between wild-type and rescue mice when the mice were compared by sex or as a whole group (**Figure 4**).

**Figure 5:**
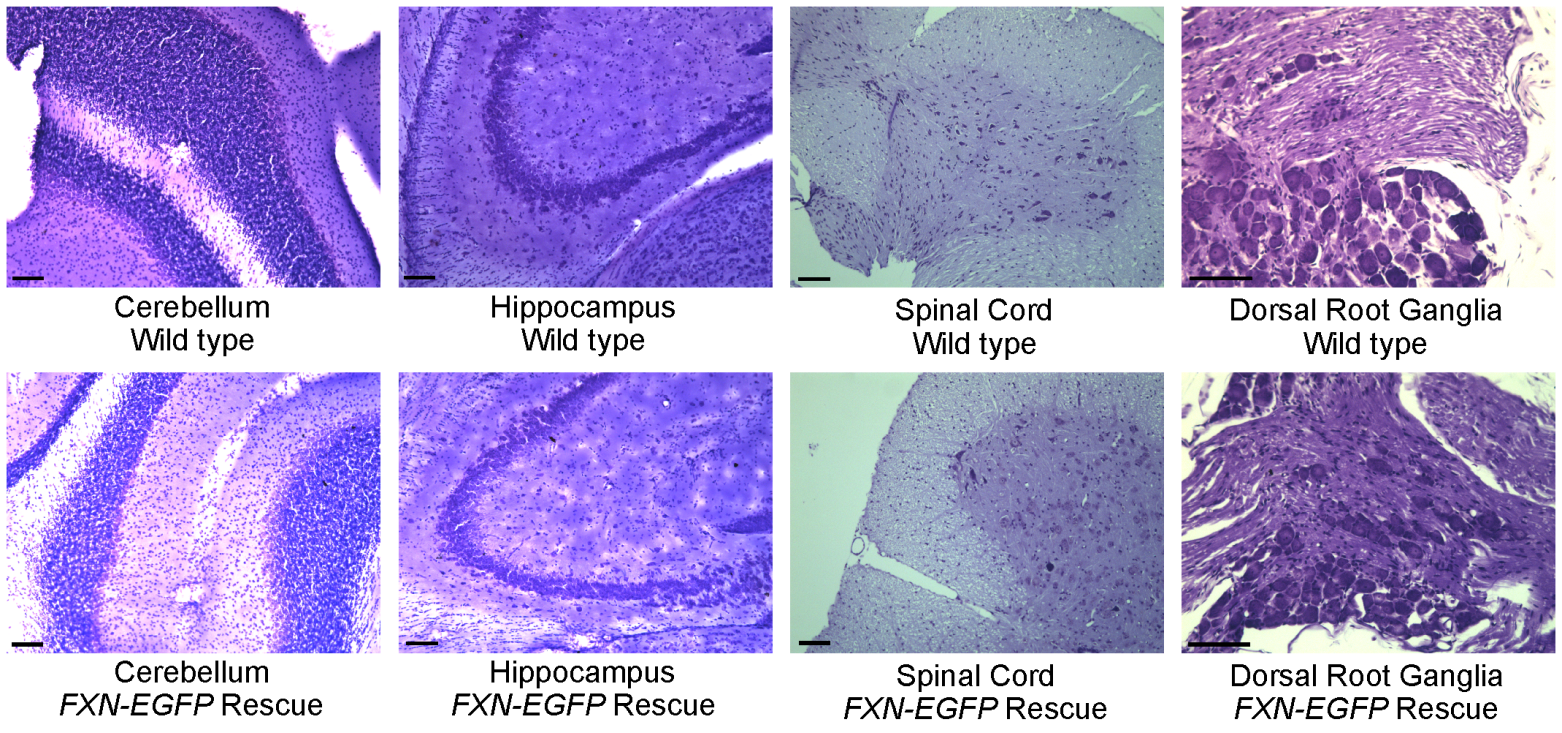
Histology. Cerebellum, hippocampus, spinal cord and dorsal root ganglia tissue samples were collected from 12 month old mice and fixed, sectioned and stained with haematoxylin and eosin. Images are representative of wild type (top row) and *FXN-EGFP* rescue mice (*FXN-EGFP* ^+^, *Fxn* ^−/−^) (bottom row). Bar in lower left of each image corresponds to 100 μm.

The same cohort of mice underwent histopathology assessment at 12 months of age. Conditional knockout and YAC-based GAA repeat expansion mouse models of FRDA [8], [35] display large vacuoles within neurons of the DRG, demyelination of large axons, lipofuscin deposition in DRG neurons and cardiomyocytes and intramitochondrial iron accumulation. Brain, spinal cord and DRG sections of the transgenic mice were qualitatively assessed for phenotypic changes associated with FRDA and compared with sections of wild type littermates. Cerebellum, hippocampus, spinal cords and DRG of transgenic and wild type animals did not demonstrate the presence of morphological changes indicative of an FRDA phenotype. Neurons in the dorsal nucleus, the anterior horn and DRG displayed no cytoplasmic vacuolation. The neuronal staining intensity was consistent across all spinal cord sections and no chromatolysis was identified. Neuronal perikaryia were generally rounded and cell nuclei were centrally located in all sections examined. No cytoplasmic or nuclear inclusion bodies were observed in the sections examined (**Figure 5**).

The *FXN-EGFP* rescue mice have been maintained for 18 generations and exhibit normal viability, fertility and life span, without any signs of abnormality. The pattern of EGFP expression throughout development in whole organs and tissues of the rescue mice paralleled that previously observed in the original *FXN-EGFP* transgenic mouse lines [29]. Cells displayed a punctate pattern of EGFP fluorescence and a lack of fluorescence in the nucleus (data not shown), indicative of the targeting of the frataxin-EGFP fusion protein to the mitochondria [25], [29].

Immunoblot analysis indicated a faint band corresponding to the size of native GFP in the *FXN-EGFP* transgenic mouse lines but there was no evidence of the presence of products corresponding to the size of native or processed frataxin protein. The apparent mitochondrial location of EGFP fluorescence in all of the *FXN-EGFP* mouse lines suggests that the frataxin-EGFP fusion protein remains largely intact. It is not possible to rule out that the *FXN-EGFP* rescue mice survive on very small amounts of unconjugated mature frataxin at levels not detectable by immunoblot, but data would indicate that the frataxin-EGFP fusion protein is a bifunctional hybrid protein able to perform the biological roles of its two component entities.

These findings have favorable implications for therapeutic approaches to FRDA that pertain to the delivery of frataxin protein conjugated to a carrier moiety. It may not be necessary to cleave the delivery component in order for frataxin to manifest normal cellular functions. The addition of 238 amino acids that constitute EGFP at the carboxyl-terminus of frataxin do not appear to severely disrupt frataxin protein function and there do not appear to be negative-dominant effects of the fusion protein.

As was the case with the original *FXN-EGFP* transgenic mouse line [29], the *FXN-EGFP* rescue mice should provide a suitable *in vivo* assay for the study of human *FXN* gene expression, the identification of long-range regulatory elements, and for the preclinical evaluation of pharmacologic inducers of *FXN* expression in a whole-animal model. These mice can also provide a useful source of cells for stem cell transplantation studies.

The original *FXN-EGFP* transgenic mouse line [29] (designated C57BL/6-Tg(FXN-EGFP)CSars) and the *FXN-EGFP* rescue mice containing the homozygous *Fxn* knockout described in this report (designated B6.129-Fxn^tm1Mkn^Tg(FXN-EGFP)CSars) are available from the Australian Phenome Bank (http://pb.apf.edu.au).
